# Comparing the mortality risks of nursing professionals with diabetes and general patients with diabetes: a nationwide matched cohort study

**DOI:** 10.1186/s12889-016-3734-1

**Published:** 2016-10-06

**Authors:** Hsiu-Ling Huang, Chuan-Yu Kung, Cheng-Chin Pan, Pei-Tseng Kung, Shun-Mu Wang, Wen-Yu Chou, Wen-Chen Tsai

**Affiliations:** 1 Department of Aged Welfare and Social Work, Toko University, Taiwan, Republic Of China; 2 Department of Public Health and Department of Health Services Administration, China Medical University, Taiwan, Republic Of China; 3 Department of Nursing, Hengchun Tourism Hospital, Ministry of Health and Welfare, Taiwan, Republic Of China; 4 Department of Urology, Hengchun Tourism Hospital, Ministry of Health and Welfare, Taiwan, Republic Of China; 5 Department of Healthcare Administration, Asia University, Taichung, Taiwan Republic Of China; 6 Department of Health Services Administration, China Medical University, 91, Hsueh-Shih Road, Taichung, Taiwan 40402 Republic Of China

**Keywords:** Nursing health education, Occupational health, Nurse with diabetes, Mortality risk, National Health Insurance, Cohort study

## Abstract

**Background:**

Nursing professionals have received comprehensive medical education and training. However, whether these medical professionals exhibit positive patient care attitudes and behaviors and thus reduce mortality risks when they themselves are diagnosed with chronic diseases is worth exploring. This study compared the mortality risks of female nurses and general patients with diabetes and elucidated factors that caused this difference.

**Methods:**

A total of 510,058 female patients newly diagnosed with diabetes between 1998 and 2006 as recorded in the National Health Insurance Research Database were the participants in this study. Nurses with diabetes and general population with diabetes were matched with propensity score method in a 1:10 ratio. The participants were tracked from the date of diagnosis to 2009. The Cox proportional hazards model was utilized to compare the mortality risks in the two groups.

**Results:**

Nurses were newly diagnosed with diabetes at a younger age compared with the general public (42.01 ± 12.03 y vs. 59.29 ± 13.11 y). Nevertheless, the matching results showed that nurses had lower mortality risks (HR: 0.53, 95 % CI: 0.38–0.74) and nurses with diabetes in the < 35 and 35–44 age groups exhibited significantly lower mortality risks compared with general patients (HR: 0.23 and 0.36). A further analysis indicated that the factors that influenced the mortality risks of nurses with diabetes included age, catastrophic illnesses, and the severity of diabetes complications.

**Conclusion:**

Nurses with diabetes exhibited lower mortality risks possibly because they had received comprehensive medical education and training, may had more knowledge regarding chronic disease control and change their lifestyles. The results can serve as a reference for developing heath education, and for preventing occupational hazards in nurses.

## Background

According to statistics provided by the International Diabetes Federation (IDF), approximately 3.8 million people die of diabetes-related diseases annually in the world [1]. In Taiwan, a marked increase has been observed in the female population diagnosed with diabetes, which has been the third leading cause of death for Taiwanese women since 2003 [2]. Type 2 diabetes is one of the most prominent chronic diseases in the world [3], and no cure has been identified. Patients with Type 2 diabetes must rely on long-term treatment and care, and complications are common. However, mortality rates and the occurrence of severe complications can be minimised if sufficient daily care is maintained [4].

There are many studies related to health-seeking and illness behaviors, the majority of which focus on specific types of patients [5, 6]. The health-belief model, the behavior model of health service utilization, the general theory of health-seeking, and the Andersen behavioral model all describe the basic foundation for the determinants of various diseases. A limited number of studies has examined the behavior of disease-affected health care providers to determine whether the influence on their performance-related behavior by their medical knowledge has been investigated [7, 8]. Nursing professionals have received comprehensive medical education and training, and therefore, they play a vital role in health care [9]. However, whether these medical professionals exhibit positive patient care attitudes and behaviors and thus reduce mortality risks when they themselves are diagnosed with chronic diseases is worth exploring.

Implemented since 1995, the National Health Insurance (NHI) is a state-run, mandatory single-payer insurance system in Taiwan. As of 2012, 99.85 % of the Taiwanese population was covered by the system [10]. The NHI covers all prescription medication, examination, and treatment administered during outpatient, inpatient, and emergency visits. The NHI database holds the medical information of all insured patients, including the treatment records for chronic diseases such as diabetes [11, 12].

In this study, the NHI Research Database (NHIRD) was employed to compare mortality risks in nursing professionals with diabetes and general patients with diabetes and identify factors that resulted in this difference. The results were used to explore whether treatment effectiveness was affected by the medical education the nursing professionals with diabetes had received and whether the knowledge they possessed reduced the relative mortality risk. The findings of this study can serve as a reference for relevant units to promote health knowledge and the health education regarding chronic diseases.

## Methods

### Setting and study population

A retrospective cohort study was conducted based on secondary data obtained from the NHIRD, and the study population was nursing professionals and general patients newly diagnosed with diabetes between 1998 and 2006. Furthermore, the participants were tracked from the date of diagnosis to death, and surviving participants were observed until December 31, 2009.

For the nursing professionals identification, we used the registry for medical personnel (PER) from the NHI. We include participant who already had been a nursing professional when she was newly diagnosed with diabetes. The nursing professionals examined in this study were registered nurses and licensed practical nurses that were registered or licensed before December 31, 2009; nursing professionals that were diagnosed with diabetes before they were licensed or registered were excluded from this study. Patients that were not registered or licensed as nursing or medical professionals (such as physical therapists, nutritionists, dentists, and physicians etc.) by December 31, 2009 were considered general patients in this study. Participants that died during the observation period and were therefore withdrawn from the NHI system (hence no additional treatment information can be obtained) were defined as deaths [13].

In consideration of the fact that nursing professionals are predominantly female (approximately 98.92 % [14]), we used female patients newly diagnosed with diabetes as the participants. For a comparison, we used a propensity score matching method as a control for age, monthly salary, urbanization of residence, catastrophic illnesses, CCI, and DCSI at a 1:10 ratio (nursing professionals with diabetes: general patients with diabetes) to account for a selection bias and to obtain two groups of participants that exhibited no statistical differences in their demographic data or health status (Table 1).

**Table 1:** Patient demographics before and after propensity score (PS) matching

Variables		Before PS matching							After PS matching (10:1)						
		Total		General patients		Nurse		*P*-value	Total		General patients		Nurse		*P*-value
		*N*	%	*N*	%	*N*	%		*N*	%	*N*	%	*N*	%	
Total patients		518,058	100.00	516,100	99.62	1958	0.38		18,601	100.00	16,910	90.91	1691	9.09	
Age								<0.001							0.928
	< 25	3092	0.60	2913	0.56	179	9.14		1194	6.42	1083	6.40	111	6.56	
	25–34	15,361	2.97	14,951	2.90	410	20.94		3214	17.28	2933	17.34	281	16.62	
	35–44	53,986	10.42	53,436	10.35	550	28.09		5568	29.93	5069	29.98	499	29.51	
	45–54	127,935	24.70	127,351	24.68	584	29.83		6059	32.57	5494	32.49	565	33.41	
	55–64	136,540	26.36	136,363	26.42	177	9.04		1894	10.18	1717	10.15	177	10.47	
	≧65	181,144	34.97	181,086	35.09	58	2.96		672	3.61	614	3.63	58	3.43	
Average age (Mean, Std)		59.22	13.15	59.29	13.11	42.01	12.03		44.14	11.86	44.19	11.87	43.60	11.78	
Monthly salary (NT$)								<0.001							0.659
	Low-income household	5010	0.97	5009	0.97	1	0.05		3	0.02	2	0.01	1	0.06	
	≦17,280	35,033	6.77	34,873	6.77	160	8.17		1826	9.82	1670	9.88	156	9.23	
	17,281 ~ 22,800	295,781	57.20	295,390	57.34	391	19.97		4312	23.18	3922	23.19	390	23.06	
	22,801 ~ 28,800	77,247	14.94	77,044	14.96	203	10.37		2011	10.81	1819	10.76	192	11.35	
	28,801 ~ 36,300	30,974	5.99	30,745	5.97	229	11.70		2015	10.83	1829	10.82	186	11.00	
	36,301 ~ 45,800	33,977	6.57	33,470	6.50	507	25.89		4128	22.19	3739	22.11	389	23.00	
	45,801 ~ 57,800	19,896	3.85	19,584	3.80	312	15.93		2540	13.66	2311	13.67	229	13.54	
	≧57,801	19,206	3.71	19,051	3.70	155	7.92		1766	9.49	1618	9.57	148	8.75	
	Missing data	934		934											
Urbanization of residence area								<0.001							0.181
	Level 1	141,455	27.35	140,779	27.33	676	34.53		6593	35.44	5998	35.47	595	35.19	
	Level 2 & 3	231,299	44.73	230,365	44.72	934	47.70		9149	49.19	8334	49.28	815	48.20	
	Level 4 & 5	94,836	18.34	94,589	18.36	247	12.61		2145	11.53	1944	11.50	201	11.89	
	Level 6 & 7	49,533	9.58	49,432	9.60	101	5.16		714	3.84	634	3.75	80	4.73	
	Missing data	935		935											
Catastrophic illnesses								0.318							0.158
	No	503,285	97.15	501,375	97.15	1910	97.55		18,200	97.84	16,554	97.89	1646	97.34	
	Yes	14,773	2.85	14,725	2.85	48	2.45		401	2.16	356	2.11	45	2.66	
Moderate to severe kidney disease								0.218							0.085
	No	440,556	85.04	438,871	85.04	1685	86.06		16,425	88.30	14,954	88.43	1471	86.99	
	Yes	77,502	14.96	77,229	14.96	273	13.94		2176	11.70	1956	11.57	220	13.01	
CCI								<0.001							0.247
	0	8776	1.69	8751	1.70	25	1.28		233	1.25	212	1.25	21	1.24	
	1 ~ 3	129,044	24.91	128,410	24.88	634	32.38		6438	34.61	5881	34.78	557	32.94	
	4 ~ 6	148,079	28.58	147,421	28.56	658	33.61		6130	32.96	5581	33.00	549	32.47	
	7 ~ 9	125,612	24.25	125,197	24.26	415	21.20		3858	20.74	3493	20.66	365	21.58	
	≧10	106,547	20.57	106,321	20.60	226	11.54		1942	10.44	1743	10.31	199	11.77	
Average CCI (Mean, Std)		6.33	3.78	6.33	3.78	5.41	3.39		5.18	3.29	5.15	3.28	5.43	3.42	
DCSI								<0.001							0.438
	0	359,786	69.45	358,301	69.42	1485	75.84		14,358	77.19	13,075	77.32	1283	75.87	
	1	79,434	15.33	79,144	15.34	290	14.81		2737	14.71	2480	14.67	257	15.20	
	2	52,368	10.11	52,232	10.12	136	6.95		1118	6.01	1009	5.97	109	6.45	
	≧3	26,470	5.11	26,423	5.12	47	2.40		388	2.09	346	2.05	42	2.48	
Average DCSI (Mean, Std)		0.54	0.98	0.54	0.98	0.37	0.76		0.34	0.73	0.34	0.73	0.36	0.75	

*Abbreviations*: *CCI* Charlson Comorbidity Index, *DCSI* Diabetes Complications Severity Index, *PS* propensity score

It’s 30 New Taiwan Dollar (NT$) per US dollar

Urbanization level of residence area (overall 7 levels; Level 1 was the most urbanized)

The *p* values less than 0.05 was considered statistically significant

To protect the confidentiality of the participants, we removed identification numbers from the data; individual participants were thus unidentifiable. The study was approved by the Institutional Review Board of the China Medical University and Hospital (IRB No.: CMUH 20130326C).

### Study design

In this study, newly diagnosis was considered to refer to patients who had been diagnosed with diabetes as a primary or secondary diagnosis (ICD-9-CM: 250 or A-code: A181) and had made 3 or more clinic visits or been hospitalized at least once within 365 days [15]. Patients that had received dialysis for less than 90 days following diagnosis or were younger than 20 or older than 90 years of age were excluded from the study. In addition, patients with Type 1 diabetes (ICD-9-CM: 6488), gestational diabetes (ICD-9-CM: 7751), neonatal diabetes (ICD-9-CM: 7902), and impaired glucose tolerance (ICD-9-CM: 6480) were also excluded.

Demographic data variables were sex, age, urbanization level of residence area (overall 7 levels; Level 1 was the most urbanized [16]), socioeconomic status (determined by the insured monthly salary). Presence of catastrophic illnesses or injuries were defined by National Health Insurance Administration in Taiwan, including 30 categories of major illnesses (e.g., stroke, hemophilia, type I diabetes, end-stage renal disease, cancer, autoimmune diseases, congenital factor disorder, Chronic Mental Illness etc.) [17]. In this study, presence of catastrophic illness was classified as yes or no. According to Deyo et al. [18], the Charlson comorbidity index (CCI) involves 17 comorbidities weighted based on severity. In addition, the definition of diabetes complication severity index (DCSI) developed by Young et al. [19] was used, and complications observed upon diagnosis or prior to the last day of observation were identified.

### Statistical analyses

Statistical Analysis System Version 9.3 was employed and chi-square tests were conducted to compare mortality rates in nursing professionals and general patients diagnosed with diabetes. The Cox proportional hazards model was utilized to compare the relative mortality risks between the two groups when all of the other variables were controlled. The model was used to identify factors that affect mortality risks in nursing professionals diagnosed with diabetes. Hazard ratios (HRs) and 95 % CIs were derived from Cox proportional hazards models. In this study, the *p* values less than 0.05 was considered statistically significance.

## Results

### Participants demographics

The number of female patients that were newly diagnosed with diabetes between 1998 and 2006 and satisfied the participant inclusion criteria was 518,058 (Table 1). Propensity score matching was performed at a 1:10 ratio to control for selection bias, yielding a sample of 18,601 (nursing professionals with diabetes vs. general patients with diabetes = 1691 vs. 16,910). The participants were tracked until the end of 2009 and the average tracking period was 6.73 ± 2.61 years (nursing professionals with diabetes vs. general patients with diabetes = 6.80 ± 2.60 y vs. 6.72 ± 2.61 y).

Before propensity score matching, among the population of female patients that were newly diagnosed with diabetes and who satisfied the participant selection criteria, significant differences were observed between the two groups in age, monthly salary, the level of urbanization of their residence, CCI, and DCSI (*P* <0 .05). Regarding age, the nursing professionals newly diagnosed with diabetes were younger on average than the general patients (42.01 ± 12.03 y vs. 59.29 ± 13.11 y). The monthly salaries of the nursing professionals were generally higher than those of the general public; 49.74 % of the nursing professionals had monthly salaries higher than NT$36,301, whereas only 10.3 % of the general patients had salaries at or above that level. Regarding the CCI and DCSI, the average CCI (5.41 ± 3.39) and DCSI (0.37 ± 0.76) of the nursing professionals were lower than those of the general patients, indicating that compared with the general public diagnosed with diabetes, the nursing professionals were healthier when newly diagnosed with diabetes (Table 1).

After the propensity score matching, there were no significant differences in the covariates between the two groups (*P* > 0.05).

### Relative mortality risks in nursing patients with diabetes and general patients with diabetes

A bivariate analysis regarding the survival of nursing professionals and general patients diagnosed with diabetes (Table 2) showed that compared with the nursing professionals, the general patients exhibited a higher mortality rate as of December 31, 2009, when the observation period ended (4.45 % vs. 2.19 %) and the difference reached statistical significance (*P* < 0.05).

**Table 2:** Bivariate analysis of participant survival

Variables		Total		Survival		Death		*P*-value
		*N*	%	*N*	%	*N*	%	
Total patients		18,601	100.00	17,812	95.76	789	4.24	
Nurses or general patients								<0.001
	General	16,910	90.91	16,158	95.55	752	4.45	
	Nurses	1691	9.09	1654	97.81	37	2.19	
Age								<0.001
	< 25	1194	6.42	1173	98.24	21	1.76	
	25–34	3214	17.28	3134	97.51	80	2.49	
	35–44	5568	29.93	5386	96.73	182	3.27	
	45–54	6059	32.57	5864	96.78	195	3.22	
	55–64	1894	10.18	1763	93.08	131	6.92	
	≧65	672	3.61	492	73.21	180	26.79	
Average age (Mean, Std)		44.14	11.86	43.73	11.49	53.27	15.83	
Monthly salary (NT$)								<0.001
	≦17,280	1829	9.83	1752	95.79	77	4.21	
	17,281 ~ 22,800	4312	23.18	4135	95.90	177	4.10	
	22,801 ~ 28,800	2011	10.81	1854	92.19	157	7.81	
	28,801 ~ 36,300	2015	10.83	1948	96.67	67	3.33	
	36,301 ~ 45,800	4128	22.19	3970	96.17	158	3.83	
	45,801 ~ 57,800	2540	13.66	2454	96.61	86	3.39	
	≧57,801	1766	9.49	1699	96.21	67	3.79	
Urbanization of residence area								0.135
	Level 1	6593	35.44	6334	96.07	259	3.93	
	Level 2 & 3	9149	49.19	8740	95.53	409	4.47	
	Level 4 & 5	2145	11.53	2062	96.13	83	3.87	
	Level 6 & 7	714	3.84	676	94.68	38	5.32	
Catastrophic illnesses								<0.001
	No	17,525	94.22	16,961	96.78	564	3.22	
	Yes	1076	5.78	851	79.09	225	20.91	
CCI								<0.001
	≦3	4097	22.03	4022	98.17	75	1.83	
	4	1926	10.35	1891	98.18	35	1.82	
	≧5	12,578	67.62	11,899	94.60	679	5.40	
Average CCI (Mean, Std)		6.54	3.59	6.42	3.52	9.28	4.16	
DCSI								<0.001
	≦1	13,650	73.38	13,269	97.21	381	2.79	
	2	2713	14.59	2564	94.51	149	5.49	
	≧3	2238	12.03	1979	88.43	259	11.57	
Average DCSI (Mean, Std)		1.02	1.31	0.98	1.26	1.92	1.91	

*Abbreviations*: *CCI* Charlson Comorbidity Index, *DCSI* Diabetes Complications Severity Index, *PS* propensity score

It’s 30 New Taiwan Dollar (NT$) per US dollar

Urbanization level of residence area (overall 7 levels; Level 1 was the most urbanized)

The *p* values less than 0.05 was considered statistically significant

**Fig. 1:**
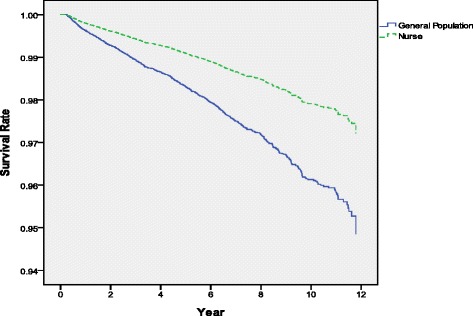
Relative mortality risk of nursing professionals with diabetes and general patients with diabetes (Cox proportional hazards model was used to control for age, monthly salary, urbanization of residence, other catastrophic illnesses, CCI and DCSI)

After controlling for other factors, Cox proportional hazards models were used to identify the mortality rate for the nursing professionals and general patients. An analysis of the data in Table 3 and Fig. 1 showed that the mortality risks of nursing professionals were lower than those of general patients (Adj. HR: 0.53, 95 % CI: 0.38–0.74). In order to compare the two groups of the same age, we further performed the stratified analyses in order to compare the two groups in terms of their mortality risk (Table 3). Regarding age, when grouped into 10-year ranges, nursing professionals younger than 35 years of age or between 35 and 44 years of age exhibited a lower mortality risk than did general patients in the same age groups (Adj. HR: 0.23 and 0.35, *P* < 0.05). Although nursing professionals older than 45 years of age had a lower mortality risk than did general patients in the same age group, this difference did not reach the level of significance (*P* > 0.05). Furthermore, whatever the CCI was, the nursing professionals had significantly lower mortality risks than did the general patients (*P* < 0.05). Regarding the DCSI, nursing professionals had lower mortality risks than did the general patients only when DCSI ≦ 1 (Adj. HR: 0.40); when DCSI >2, no significant difference was observed between the 2 groups (*P* > 0.05).

**Table 3:** Stratified analyses: relative mortality risks in nursing patients with diabetes and general patients with diabetes

Variables		General patients			Nurse			Adj. HR^a^ (Nurse: GP)	95 % CI		*P*-value
		Total (*N*)	Death (*N*)	Death (%)	Total (*N*)	Death (*N*)	Death (%)				
Total		16,910	752	4.45	1691	37	2.19	0.53	0.38	0.74	<0.001
Age											
	< 35	4016	99	2.47	392	2	0.51	0.23	0.06	0.91	0.037
	35–44	5069	176	3.47	499	6	1.20	0.35	0.16	0.80	0.012
	45–54	5494	185	3.37	565	10	1.77	0.57	0.30	1.08	0.085
	55–64	1717	125	7.28	177	6	3.39	0.51	0.22	1.15	0.106
	≧65	614	167	27.20	58	13	22.41	0.91	0.51	1.60	0.735
CCI											
	< 6	7370	154	2.09	700	4	0.57	0.29	0.11	0.78	0.014
	≧6	9540	598	6.27	991	33	3.33	0.59	0.41	0.83	0.003
DCSI											
	≦1	12,358	364	2.95	1292	17	1.32	0.40	0.25	0.65	<0.001
	2	2491	141	5.66	222	8	3.60	0.70	0.34	1.44	0.334
	≧3	2061	247	11.98	177	12	6.78	0.72	0.40	1.29	0.267

^a^The Cox proportional hazards models have controlled for age, monthly salary, urbanization of residence, other catastrophic illnesses, CCI and DCSI

### Factors that affect the mortality of nursing professionals with diabetes

As shown in Table 4, factors that affected the mortality of nursing professionals with diabetes included age, catastrophic illnesses, and DCSI. The mortality risk of nursing professionals 65 years of age or older was 23.54 times that of nursing professionals younger than 35 years of age (95 % CI: 5.02–110.33). The mortality risk of nursing professionals with catastrophic illnesses was 6.90 times that of those without such conditions (95 % CI: 2.92–16.29). In addition, the mortality risk of nursing professionals with a DCSI ≧ 3 was 2.72 times that of those with a DCSI ≦ 1 (95 % CI: 1.20–6.16).

**Table 4:** Factors that affect the mortality of nursing professionals with diabetes

Variables		Unadj. HR	*P*-value	Adj. HR	95 % CI		*P*-value
Age							
	< 35 (reference)						
	35–44	2.28	0.313	1.79	0.35	9.18	0.487
	45–54	3.60	0.098	2.76	0.58	13.13	0.202
	55–64	6.93	0.018	3.31	0.63	17.36	0.157
	≧65	48.29	<0.001	23.54	5.02	110.33	<0.001
Monthly salary (NT$)							
	≦17,280 (reference)						
	17,281 ~ 22,800	0.50	0.219	0.98	0.30	3.17	0.967
	22,801 ~ 28,800	1.10	0.856	1.37	0.45	4.21	0.579
	28,801 ~ 36,300	0.52	0.308	1.01	0.26	3.98	0.989
	36,301 ~ 45,800	0.31	0.050	0.75	0.21	2.73	0.667
	45,801 ~ 57,800	0.43	0.194	0.58	0.15	2.19	0.422
	≧57,801	0.35	0.202	0.45	0.09	2.39	0.351
Urbanization of residence area							
	Level 1 (reference)						
	Other Level	0.79	0.476	1.14	0.56	2.33	0.724
Catastrophic illnesses							
	No (reference)						
	Yes	5.82	<0.001	6.90	2.92	16.29	<0.001
CCI							
	< 5 (reference)						
	≧5	4.61	0.011	1.29	0.37	4.55	0.688
DCSI							
	≦1 (reference)						
	2	2.70	0.021	2.20	0.91	5.27	0.078
	≧3	4.79	<0.001	2.72	1.20	6.16	0.017

*Abbreviations*: *CCI* Charlson Comorbidity Index, *DCSI* Diabetes Complications Severity Index, *PS* propensity score

It’s 30 New Taiwan Dollar (NT$) per US dollar

Urbanization level of residence area (overall 7 levels; Level 1 was the most urbanized)

The *p* values less than 0.05 was considered statistically significant

## Discussion

In this study, utilizing the Cox proportional hazards model indicated (Table 3) that the nursing professionals with diabetes had lower mortality risks compared with the general patients with diabetes (Adj. HR: 0.53). Using the propensity score matching, the nursing professionals’ cohort and the general patients’ control group were similar in terms of demographics, health status, and their socio-economic status (P > 0.05). The two groups may have differed in terms of their knowledge, their attitude, and their practice of health care. We found that nurses had lower mortality risks possibly because nursing professionals have more medical knowledge, which was consistent with the results of previous studies with physicians [8]. Nursing professionals diagnosed with diabetes play a dual role as both providers and recipients of health care. Due to the health care knowledge that they possess, nursing professionals are more likely to adopt active and positive health care attitudes when diagnosed with chronic diseases. In addition, they are more capable of adjusting their lifestyles, and therefore, they have lower mortality risks compared with general patients that are diagnosed with diabetes. This finding confirmed the knowledge, attitude, and practice theory [20–22].

On an average, nursing professionals suffered from newly onset diabetes at a younger age than general patients (42.01 ± 12.03 y vs. 59.29 ± 13.11 y), possibly because nursing professionals have to work in shifts. Niedhammer et al. [23] found that nursing professionals who worked in night shifts were more likely to gain weight and become overweight. Other studies [24, 25] have also discovered that working shifts could result in an increased risk of metabolic diseases. Timothy et al. [26] claimed that shift work was a risk factor for diabetes.

However, when the nurses and the general patients were diagnosed with diabetes, we found that the nursing professionals were healthier than the other women in terms of their DCSI score (0.37 ± 0.78 vs. 0.54 ± 0.98, Table 1), which suggests a screening bias; either the nurses received the diagnosis prematurely and/or the others received it later, because the awareness of the disease could be more pronounced among the nurses due to their medical knowledge. In addition, the nurses could be more aware of the importance of preventive strategies once the disease has been diagnosed, giving them a better prognosis as compared to the others, e.g., lower mortality risks in the younger age strata.

After we used propensity score matching, the nursing professionals with diabetes had no significant difference that was observed between mortality risks in diabetic nursing professionals and general patients above 45 years of age (Table 3), possibly because of a familial tendency or that combined with environmental influences [27]. This is also possibly because the capability to adjust lifestyles decreases with age. In addition, knowledge cannot always affect attitude nor can positive patient care (practice) always be performed [28, 29].

An analysis regarding factors that influence mortality in nursing professionals diagnosed with diabetes (Table 4) showed that nursing professionals that were older, had catastrophic illnesses, or had high DCSI exhibited higher mortality risks. The results were consistent with those of previous studies [30, 31]. The analysis of monthly salaries indicated that this variable had no significant influence on the mortality risks in nursing professionals with diabetes. This result disagreed with that of Kposowa [32], who investigated the relationship between financial status and mortality by examining 527,426 U.S. patients with diabetes. Kposowa discovered that financial status and income were crucial factors that affected mortality in patients with diabetes. Specifically, people with a lower financial status exhibited higher mortality rates. In this study, monthly salary had no significant influence on the mortality risks in nursing professional with diabetes possibly because all nursing professionals had similar levels of medical education and patient care knowledge; therefore, the approaches and quality of care they received were similar. Consequently, although nursing professionals with higher monthly salaries had relatively low mortality risks, this difference did not reach the level of significance.

### Limitations

The study has the following several limitations: (1) only data from the NHIRD were examined, and factors such as the lifestyles and health behavior of the patients were not considered. Therefore, we used the propensity score matching to avoid a selection bias from confounding variables in observational studies and imitate the results of a randomized controlled trial [33]. The longitudinal database provides a better opportunity for accumulation of data concerning nursing professionals with diabetes and survival analyses; and (2) no clinical data were obtained to verify the accuracy of the diabetes classification in the NHIRD, where the International Classification of Diseases-9 codes were applied. To overcome the second limitation, we adopted rigorous classification criteria: patients with diabetes were defined as people that had been diagnosed with diabetes during primary or secondary diagnosis (ICD-9-CM: 250 or A-code: A181) and had made 3 or more clinic visits or been hospitalized at least once within 365 days [15].

In this study, because each nurse’s number of years of service and shift lengths was unknown, the correlation between their shift work and diabetes mortality risk could not be determined, which is another limitation.

## Conclusion

This study is the first attempt to use NHIRD data for analyzing relative mortality risks in nursing professionals with diabetes and general patients with diabetes. Patients with Type 2 diabetes treatment would not leave their medical care service insurance, especially under a universal health insurance program. The results showed that although nursing professionals were diagnosed with diabetes at younger ages, they had lower mortality risks compared with general patients with diabetes in their age groups. Health professionals may have more medical knowledge regarding earlier screening and disease control and prevention than others. The nursing professionals may use their own professional knowledge to engage in healthy lifestyles in a way that reduces their risk of illness.

Nursing professionals working at clinics must deal with heavy workloads and shift work and are therefore prone to occupational hazards. Stress and shift work are risk factors for diabetes. The results of this study can serve as a reference for understanding the occupational hazards encountered by nursing professionals and for devising plans for improving the health of nursing professionals. In addition, the results also can serve as a reference for developing heath education.

## Abbreviations

CCI: Charlson Comorbidity Index
CI: Confidence interval
DCSI: Diabetes complication severity index
HR: Hazard ratio
ICD: International Statistical Classification of Diseases
IDF: International Diabetes Federation
NHI: National Health Insurance
NHIRD: NHI Research Database
NT$: New Taiwan Dollar
PS: Propensity score
US: United States
